# Bilateral adrenal haemorrhage with renal infarction after ChAdOx1 nCoV-19 AstraZeneca vaccination

**DOI:** 10.1259/bjrcr.20210139

**Published:** 2022-01-10

**Authors:** Tun Tha, Iana Martini, Elena Stefan, Sridhar Redla

**Affiliations:** 1 Sandwell and West Birmingham Hospitals NHS Trust, Birmingham, UK; 2 The Princess Alexandra Hospital NHS Trust, Harlow, UK

## Abstract

Vaccine-induced thrombotic thrombocytopaenia (VITT) is a rare syndrome associated with the ChAdOx1 nCoV-19 (AstraZeneca) vaccine. We detail a case of vaccine-induced thrombotic thrombocytopaenia in a 47-year-old female who was found to have bilateral adrenal haemorrhage, renal vein thrombosis, renal infarction and pulmonary embolism 13 days post-vaccination with ChAdOx1 nCoV-19.

## Introduction

The development of effective vaccines against severe acute respiratory syndrome coronavirus 2 (SARS-CoV-2) has represented a pivotal accomplishment in reducing the still widespread global burden of coronavirus disease 2019 (Covid-19). There has, nonetheless, been scrutiny towards the safety profile of Covid-19 vaccines since adverse thromboembolic phenomena were first reported in late February 2021^1^ and has pertained chiefly to the ChAdOx1 nCoV-19 (AstraZeneca) adenoviral vector vaccine. It has been established that the vaccine is occasionally associated with a very rare, but serious distinct clinical syndrome of atypical thromboses with low platelets, referred to as vaccine-induced thrombotic thrombocytopaenia (VITT).^1,2^

Greinacher et al demonstrated pathophysiological similarities of VITT to heparin-induced-thrombocytopaenia (HIT)^1^ in that both syndromes are mediated by platelet-activating antibodies to platelet factor 4 (PF4). High titres of PF4 antibodies were detected in VITT patients despite no prior heparin exposure and were able to activate platelets independently.^1–3^ Based on such findings, the National Institute for Health and Care Excellence issued a consensus recommendation to use an enzyme-linked immunosorbent assay (ELISA) for PF4 antibodies to reliably confirm diagnosis of VITT.^4^ Sites of thrombosis have characteristically included the cerebral venous sinuses and splanchnic veins,^5^ although thrombosis has also been found to occur in more typical sites of venous and arterial thromboembolism (*e.g.* pulmonary embolism).

## Case report

**Figure 1.**
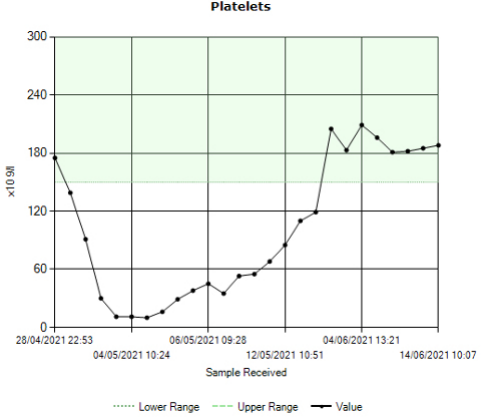
Graph illustrating trend in platelet count over the course of the patient’s hospital stay.

A 47-year-old female with no significant past medical history presented to A&E with sudden onset epigastric and central abdominal pain that had been worsening over 3 days and was intermittent in nature. The pain was reported to radiate through to the back and was exacerbated after eating, accompanied by one episode of vomiting. Clinically, the patient looked well, alert and orientated with normal vital signs. Examination revealed a non-distended soft abdomen with mild epigastric tenderness to palpation. Initial blood tests on admission showed a white cell count of 16.7 × 10^9^/L, C-reactive protein of 2 mg l^−1^, haemoglobin of 132 g l^−1^, international normalised ratio of 1.0 and an amylase of 62 IU l^−1^; the platelet count was found to be 139 × 10^9^/L (normal range 150–400 × 10^9^/L) on admission – a drop from a baseline count of 175 × 10^9^ l^−1^ (Figure 1). Alarmingly, lactate level peaked to 7 mmol l^−1^ on the day of admission, but this settled with fluid resuscitation. The patient had no discernible previous history of thromboembolism or exposure to heparin, and no family history of thromboembolic disease or hypercoagulability states, the patient did report, however, five previous spontaneous terminations which were not investigated further.

Initially, it was clinically presumed that the patient was suffering from duodenitis with a possible intra-abdominal infection, the medical team thus started intravenous Piperacillin/Tazobactam and Gentamicin as per local antibiotic guidance together with Omeprazole and analgesics. Urgent CT of the abdomen and pelvis performed was only equivocal for duodenitis and showed minimal peripancreatic fat stranding that was later correlated with a normal amylase to exclude acute pancreatitis. Oesophagogastroduodenoscopy (OGD) on Day 1 post-admission was further in keeping with mild duodenitis at the level of D1 (first part of the duodenum). Subsequent duodenal biopsies showed evidence of normal mucosa.

Over the course of 5 days post-admission however, the platelet count was observed to decline to 11 × 10^9^/L (Figure 1). Work-up for consumptive coagulopathy revealed adequate fibrinogen levels (4.2 g l^−1^) and abdominal imaging revealed no evidence of splenomegaly to explain the thrombocytopaenia.

At this point, it came to light that the patient had received the ChAdOx1 nCoV-19 vaccine 8 days prior to presenting to hospital and as such VITT became a leading differential diagnosis to work-up.

Another key finding was that the patient’s D-dimer was notably raised at 24,000 mcg/L, beyond the level typically expected for venous thromboembolism and was befitting for VITT.

**Figure 2.**
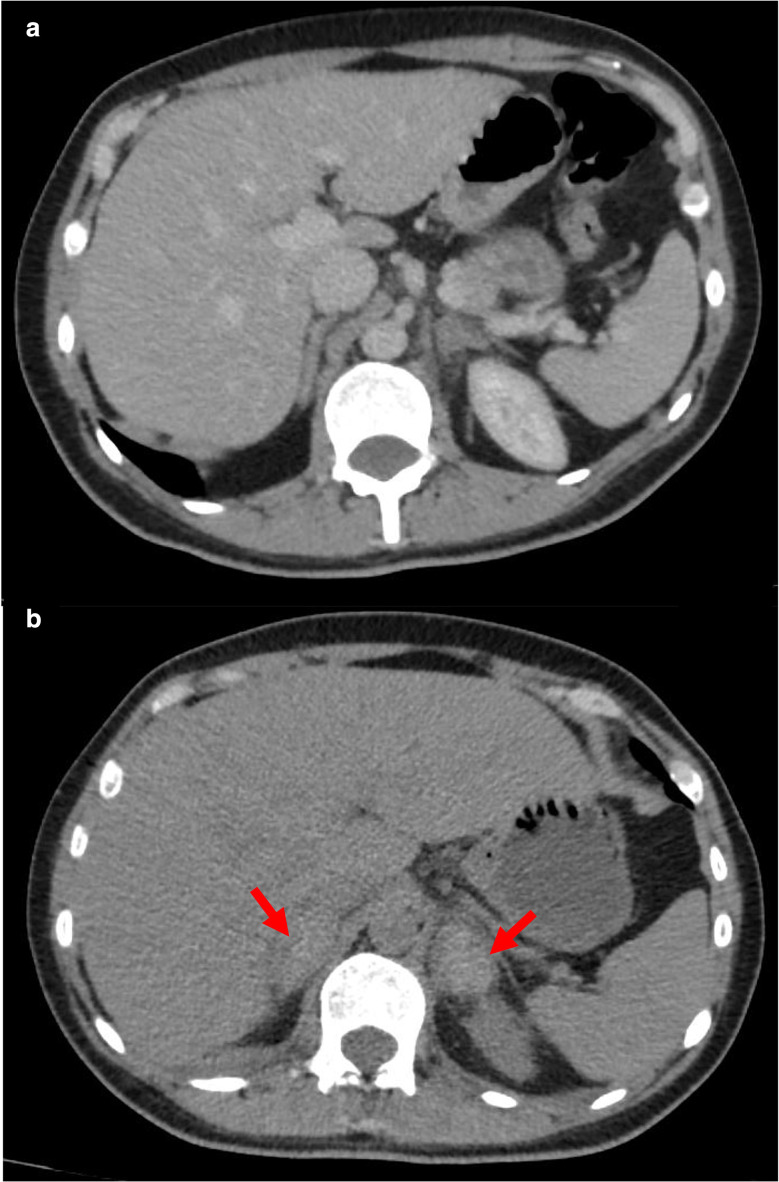
(**A**) Initial axial unenhanced CT scan of the abdomen on day of admission showing relatively unremarkable adrenal glands. (**B**) Axial unenhanced CT of the abdomen showing bilateral adrenal haematomas (arrows) Day 5 post-admission.

A triple phase CT of the abdomen and pelvis was subsequently performed on Day 5 post-admission, wherein it was found that the adrenal glands were bilaterally enlarged and demonstrated high attenuation (54 Hounsfield units) on native CT (Figure 2B), characteristic of adrenal haemorrhage, which was presumed to be due to bilateral adrenal vein thromboses. There was no evidence of a bacterial septic process at the time to suggest a diagnosis of Waterhouse-Friderichsen syndrome, with no clinical signs of sepsis or adrenal insufficiency on observations and examination.

**Figure 3.**
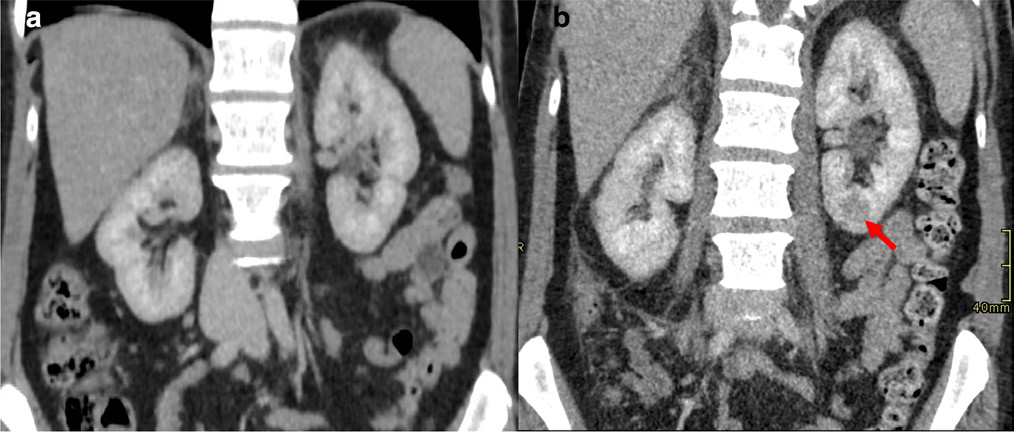
(**A**) Initial coronal CT of the abdomen on day of admission showing normal enhancement of the renal parenchyma. (**B**) Coronal CT of the abdomen showing a focus of renal cortical hypoenhancement (arrow) Day 5 post-admission.

**Figure 4.**
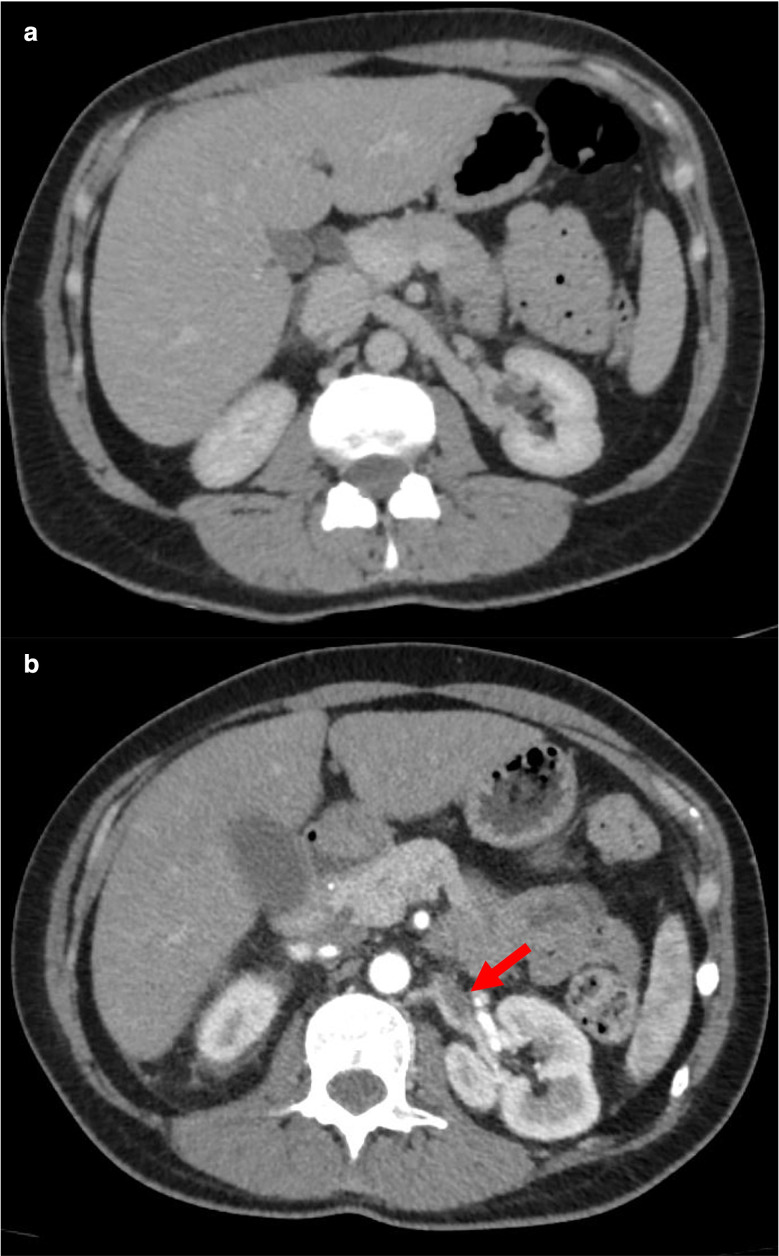
(**A**) Initial CT of the abdomen arterial phase on day of admission showing normal opacification of the renal arteries. (**B**) CT of the abdomen arterial phase showing a subocclusive filling defect in the left renal vein (arrow) with a well opacified renal artery Day 5 post-admission.

There were, furthermore, foci of hypoenhancement in the cortex visualised at both the upper and lower poles of the left kidney (Figure 3B) and lower pole of the right kidney indicative of bilateral renal infarction. A subocclusive filling defect of the left renal vein (Figure 4B) together with a subtle but possibly similar filling defect of the right renal vein were noted, with well-opacified renal arteries. It seems unlikely that such infarcts were secondary to bilateral non-occlusive renal vein thrombosis, and we suspect concomitant arterial microthrombi. The right hepatic vein was not opacified, in keeping with hepatic vein thrombosis; the inferior vena cava was patent.

**Figure 5.**
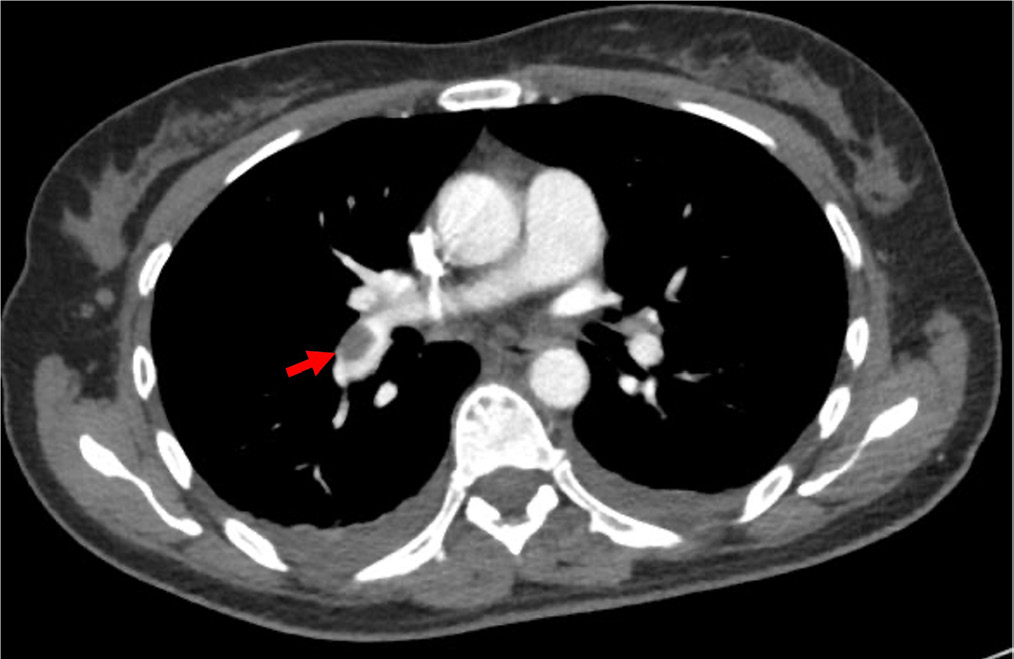
CT pulmonary angiogram demonstrating embolus in the right lower lobe pulmonary artery (arrow).

Although the patient did not report any shortness of breath, the degree of epigastric pain she exhibited was out of keeping with the OGD findings, and as such a CT pulmonary angiogram was requested to identify whether her pain was referred from lung parenchymal infarction following a pulmonary embolism; CT pulmonary angiogram subsequently demonstrated a right-sided pulmonary embolus (Figure 5).

A full prothrombotic investigation panel was performed that included screening tests for antiphospholipid syndrome in view of the previous spontaneous terminations of pregnancy, all of which came back negative. Results of an ELISA for antibodies to PF4 were positive and provided a final attestation for a definitive diagnosis of VITT.

The patient was treated in accordance with guidance from the Expert Haematology Panel of The British Society of Haematology.^6^ Urgent intravenous immunoglobin was administered followed by anticoagulation with Argatroban at an initial dose of 0.5 mcg/kg/min; intravenous hydrocortisone was given for adrenal insufficiency later confirmed by short Syncathen test. The patient was then loaded on and discharged with a direct oral anticoagulant and oral fludrocortisone, and is currently pending outpatient haematology and endocrinology follow-up.

## Discussion

Existing literature to date suggests that VITT is a clinically heterogenous phenomenon: there appears to be a wide range of possible thrombotic sites; there may be individual or multiple lesions; and these present with varied onset, duration and severity. Bilateral adrenal haemorrhage has been previously reported^7^ but seems to be a less common sequela than cerebral venous sinus thrombosis or splanchnic vein thrombosis.^8^ At the time of writing (28 May 2021), no case reports detail any renal manifestations of VITT, our case therefore emphasises the vast spectrum of thrombotic sites. It would furthermore be a matter of academic interest why certain sites are more affected than others.

Although research is still inconclusive, some initial reports note that females may be more likely to develop VITT than their male counterparts, with the majority of cases being in patients under the age of 55 years^5^. The aforementioned patient satisfies both of these proposed risk factors. VITT has been known to generally occur 5–28 days after vaccination as was true in our case.

With regards to the aetiology of the duodenitis noted on initial OGD, the Yellow Card data detailing reported side-effects and reactions to the AstraZeneca vaccine as of 10 September 2021 demonstrate that there have been two formally reported cases of duodenal ulceration and 878 cases of the vaguer entity of “dyspepsia".^9^ Although we cannot comment with full certainty, it is possible that the duodenitis may have been related to the COVID vaccine, but equally may have been an incidental finding: the epigastric discomfort the patient experienced may have simply represented referred pain related to the aforementioned pulmonary embolus, and given the normal duodenal biopsy results, the inflammation perceived may have only been of subjective value.

As highlighted similarly by other case reports,^10,11^ our case illustrates the potential acute clinical trajectory of VITT, which may initially present with non-specific symptoms—as with our patient, who originally presented with epigastric discomfort. This surreptitious facet of the disease may have implications on radiological diagnosis: indeed, we observed that an initial CT scan of the abdomen and pelvis showed no evidence of thromboembolic pathology, and it was only when repeat, contrast-enhanced cross-sectional imaging was requested 5 days later (in consideration of a dropping platelet count) that the presence of thrombosis was confirmed. This also underlines the diagnostic importance of laboratory blood counts together with radiological correlation.

Fortunately, cases of VITT are very rare and it should be stressed that the UK Medicines and Healthcare Products Regulatory Agency have maintained that the benefit of the AstraZeneca vaccine outweighs risk, particularly in patients over the age of 40 years. Nevertheless, it is still crucial for clinicians to be mindful and vigilant as the vaccine rollout continues, and this may become evermore important as younger age groups are being reached.

## Learning points

- VITT can affect a wide range of thrombotic sites, not being limited to the cerebral and splanchnic veins.
- VITT can present with non-specific symptoms and repeated radiological imaging with contrast should be considered if haematological evidence of evolving thrombosis is noted (*e.g.* declining platelet count).
- Clinicians must be vigilant of VITT as vaccination efforts increase.
